# What has been used to improve patients’ spiritual health in primary care: A realist review

**DOI:** 10.1080/13814788.2026.2739239

**Published:** 2026-10-12

**Authors:** Ishbel Orla Whitehead, Philip Mordue, Mark Adley, Elizabeth Westhead, Jabez Barnabas, Amy O’Donnell, Barbara Hanratty

**Affiliations:** a Population Health Sciences Institute, Faculty of Medical Sciences, Campus for Ageing and Vitality, Newcastle University, Newcastle upon Tyne, United Kingdom; b NIHR Information Observatory, The Catalyst, Newcastle Helix, Newcastle University, Newcastle upon Tyne, United Kingdom

**Keywords:** Spiritual health, realist review, primary care

## Abstract

**Background:**

Spiritual health is a vital aspect of holistic health linked to improved outcomes, yet little is known about approaches used in primary care to support this dimension of wellbeing.

**Objectives:**

To identify which interventions have been used to improve patients’ spiritual health in primary care, and what works, for whom, and in which contexts.

**Methods:**

A qualitative realist review was registered with PROSPERO and conducted in line with RAMESES standards. Systematic database searches were completed in May 2024 and June 2025. Iterative analysis and synthesis were informed by discussions with professionals to situate findings within real-world practice contexts.

**Results:**

Five European interventions were identified, each offering person-centred, listening-based support to address spiritual or existential concerns. These services operated in small, motivated pockets of practice rather than as routine provision. Validated outcome measures showed improvements in emotional wellbeing, but none directly assessed spiritual health. Most interventions followed a chaplaincy model adapted from hospital care and faced barriers to wider implementation: limited funding, workforce pressures, and inconsistent referral pathways.

**Conclusion:**

Spiritual health interventions can provide valuable holistic support within primary care, but current provision is fragmented, locally driven, and often unsustainable. Improving timely referrals, increasing practitioner confidence, and reducing stigma surrounding spiritual health are priorities. A social asset-based approach may complement existing practice by equipping primary care staff to recognise spiritual need and connect patients with community organisations. Further research is required to determine the acceptability, effectiveness, and scalability of chaplaincy and alternative asset-based models of spiritual care within primary care.

## KEY MESSAGES

- Spiritual health is an important part of holistic care but seldom addressed in primary care.
- Current interventions show benefits for emotional well-being, but face barriers such as fragmented provision, short-term funding, and lack of direct measures.
- Stronger community partnerships and asset-based approaches could improve access to spiritual health support.

## Introduction

General Practice aims to be an holistic medical speciality considering not just the disease, but how people and their communities can achieve healthy lives [1,2]. Spiritual health, an often-neglected aspect of holistic health and salutogenesis, includes meaning, purpose, moral values, connection, transcendence and expressions of spirituality [3,4]. Although it may include religion, it is not synonymous with it [3,4]. Understandings are culturally diverse, encompassing identity, relationships, nature, ancestry, language and collective practice [5]. We therefore use a UK-based pragmatic working definition rather than a culturally universal one.

Spiritual health is associated with physical and mental health, longevity and wellbeing, while spiritual distress may be harmful [6–9]. It may therefore support health creation and whole-person primary care, yet provision remains inconsistent [10]. Embedded biopsychosocial-spiritual care has been proposed, particularly for ageing populations with multimorbidity, but how spiritual health needs are addressed in European primary care remains unclear [10].

Chaplaincy has been introduced in some European primary care settings [11]. However, it developed largely in secondary care, has Christian roots, and may not be the only or most suitable primary care model [12]. Across Europe, religiosity varies by age, generation, population and geographical context. Declining religious affiliation in some groups coexists with continuing religious belief, religious plurality, and diverse non-religious and spiritual worldviews [13,14]; although older adults tend to report greater religiosity, successive generations have become less religious, contributing to an overall decline [14]. Contemporary chaplaincy increasingly seeks to address religious and non-religious existential needs and to support people of “all faiths and none”, but whether this inclusivity is achieved in practice remains insufficiently evaluated.

Partnerships with community faith-based organisations (CFBOs), including parish nursing, may extend support but can be hindered by unclear roles, inequalities and concerns about proselytising [15,16]. Social prescribing may connect patients with faith and non-faith community resources [17].

Proposals of ‘embedded models’ of spiritual health care appear largely conceptual and aspirational, relying on assumed benefits for quality of care and system efficiency without offering empirical evidence or clear solutions to practical barriers such as time, training, resource constraints, and pluralistic spiritual needs [10]. Before determining how spiritual health should be incorporated into holistic primary care, it is necessary to examine which interventions have been implemented in Europe, how they work, for whom and under what conditions.

## Aims and objectives

### Aims

This study therefore sought to understand what interventions, initiatives and projects have been used to improve patients’ spiritual health in primary care in Europe, what has worked, how it has been implemented, for whom and in what contexts? How do these interventions fit within primary care, and other aspects of the primary care offer?

### Objectives

 

1. To identify initiatives used in Europe to address patients’ spiritual health needs in primary care.
2. To synthesise the evidence of what has been tried, for whom, in what contexts to address spiritual health in primary care.
3. To understand what works, for whom, and in which contexts, to enhance spiritual health in primary care
4. To understand how these initiatives interact with other holistic health interventions in primary care, for example ideas such as social prescribing in the UK.

## Methods

This review followed the processes for conducting realist reviews outlined by Pawson et al. [18], with reporting following the RAMESES (Realist And MEta-narrative Evidence Syntheses: Evolving Standards) publication standards [19] (Appendix A).

A realist review was selected because the review addressed an explanatory question rather than seeking only to map the range and characteristics of the literature [20]. Spiritual health interventions were expected to be heterogeneous and their effects are likely to depend on cultural, religious, social and service contexts. Realist synthesis is a theory-driven approach [21] that seeks to explain how outcomes are generated through interactions between context and mechanism [18–20,22]. It therefore enabled each intervention to be examined within its real-world setting and supported the development and refinement of programme theories about what works, for whom, through which mechanisms, and under what conditions [18,20,21].

Before the formal systematic search, broad, non-systematic exploratory searches were undertaken to establish the presence and range of relevant literature, identify terminology and potential seed papers, and inform the scope and development of the database search strategy [18–20]. The subsequent systematic searches were developed with input from an information scientist (JB). Search strategies were discussed with the expert advisory group and tested with seeding studies that they had identified. Stakeholders, including patients and the public, have been included in this project from inception, *via* Voice (a network of public, patients, and carers who contribute to research) [23] and the SHARP (Spiritual Health Awareness and Recommendations in Primary care) project advisory group. The initial programme theory (Figure 1) was discussed and developed within the research team, at a national UK conference for primary care research [24], and with expert advisory groups.

**Figure 1.**
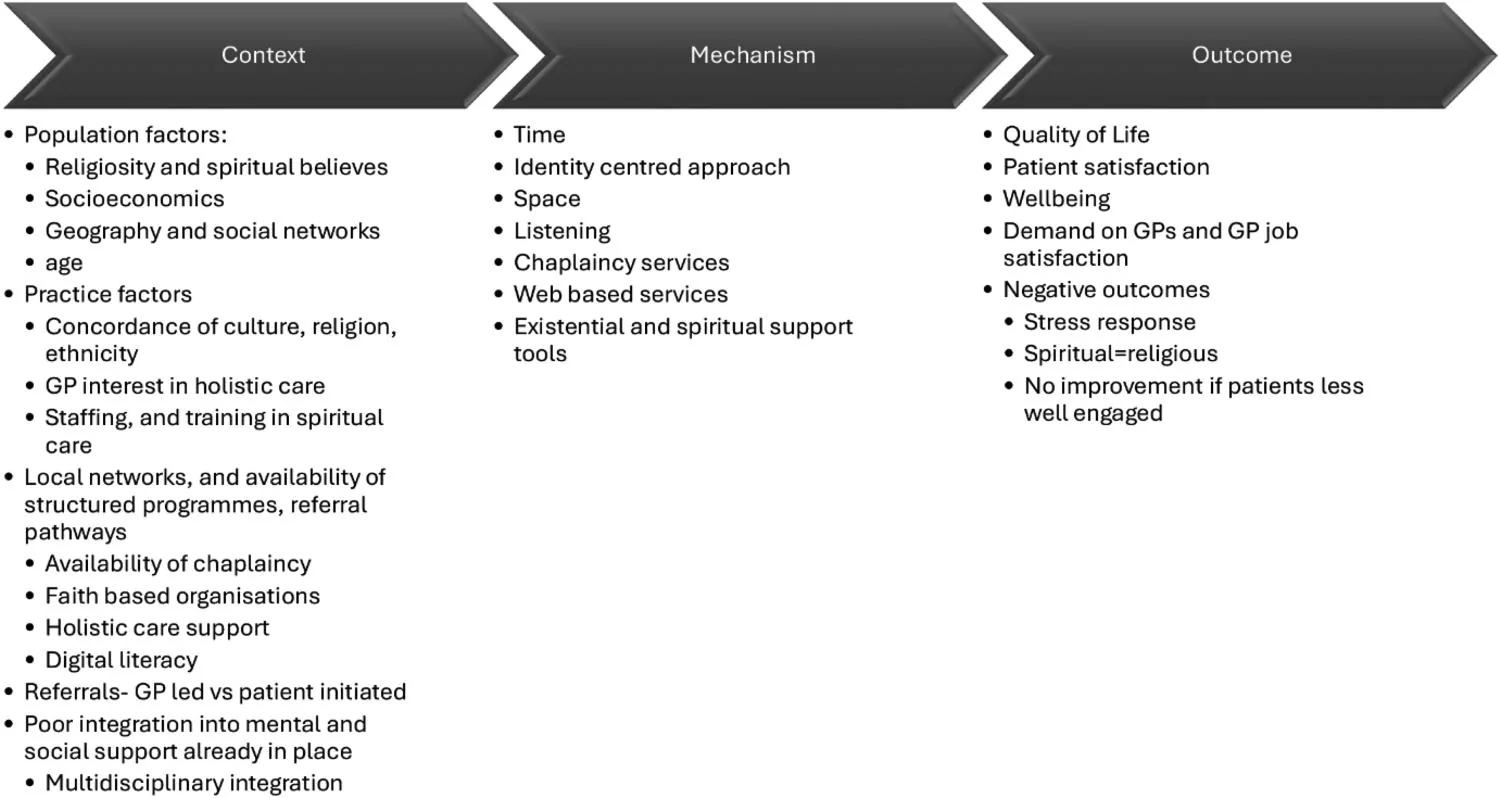
Initial programme theory.

Initial searches identified substantial international literature, including work with First Nations, Māori and other Indigenous populations [25–30]. There is a known dominance of the USA in this topic [31–34]. We restricted the synthesis to Europe to compare interventions within broadly similar cultural and health-system contexts and within the team’s expertise, while recognising the wider global literature.

Spiritual health is a broad concept, as diverse as people themselves. In this work, spiritual health was not defined and left to the participant to define themselves. The authors use a definition of spiritual health derived from UK General Practitioners and further developed with social prescribers: self-actualisation, peace, purpose and meaning; transcendence, connectivity and relationships beyond the self; and expressions of spirituality [3,4]. A working definition of spiritual health [3,4] informed the search and screening process but was applied inclusively. Studies were retained at title and abstract screening where spirituality, or related concepts such as meaning, purpose, connection, holistic health, wellbeing or salutogenesis, might form part of the intervention; final eligibility was determined through full-text assessment of the intervention’s aims, content and intended outcomes.

Box 1 Inclusion and exclusion criteria for the realist review

**Inclusion criteria:**

- All study types where *something* had been tried with patients (an intervention)
- Setting includes primary care
- Any initiative targeting spiritual health—either explicitly (spirituality, religious health, or related terms) or by addressing aspects of spiritual health.

**Exclusion criteria:**

- Training interventions that did not include patients
- Studies conducted entirely outside primary care, with no primary care involvement, collaboration or referral pathway. (e.g. community mental health teams, churches)
- Interventions explicitly addressing mental or physical health only

An iterative process was undertaken, with searches, appraisal, and synthesis taking place simultaneously [18–20,35]. Informal searches and concept mining were used to inform initial theories [20,36] and get a feel for the literature. Formal systematic searches were developed with an information scientist (JB) and conducted in MEDLINE, Embase, PsycINFO and CINAHL. The original searches covered publications from 2000 to 24 May 2024 and were subsequently updated to 25^th^ June 2025. Full database-specific strategies, including controlled vocabulary, free-text terms, field codes, Boolean combinations and date limits, are provided in Appendix B. Forwards and backwards citation searching, reference checking, and contacting authors or stakeholders supplemented the database searches. Additional data were sourced through further evidence searches and ongoing concept mining.

Systematic searches yielded 8003 individual articles that were screened by four researchers (IOW, PM, EW, and MA), with 1863 (23%) double screened (Figure 2). An additional six articles were identified through other sources. A total of 582 full-text articles were assessed for eligibility (see Box 1). After detailed appraisal of seventeen articles by IOW, PM, and MA, four were excluded. Selection decisions were discussed throughout screening and appraisal. Where eligibility was uncertain at title and abstract screening, records were retained for full-text assessment. Any remaining uncertainty at full-text review or data extraction was discussed by the wider review team, and inclusion or exclusion was agreed collectively. Interventions were mapped, but our focus was on European interventions. Interventions that did not evaluate the impact on patients (for example, staff only training with no evaluation) were excluded but mapped to allow understanding of context.

**Figure 2.**
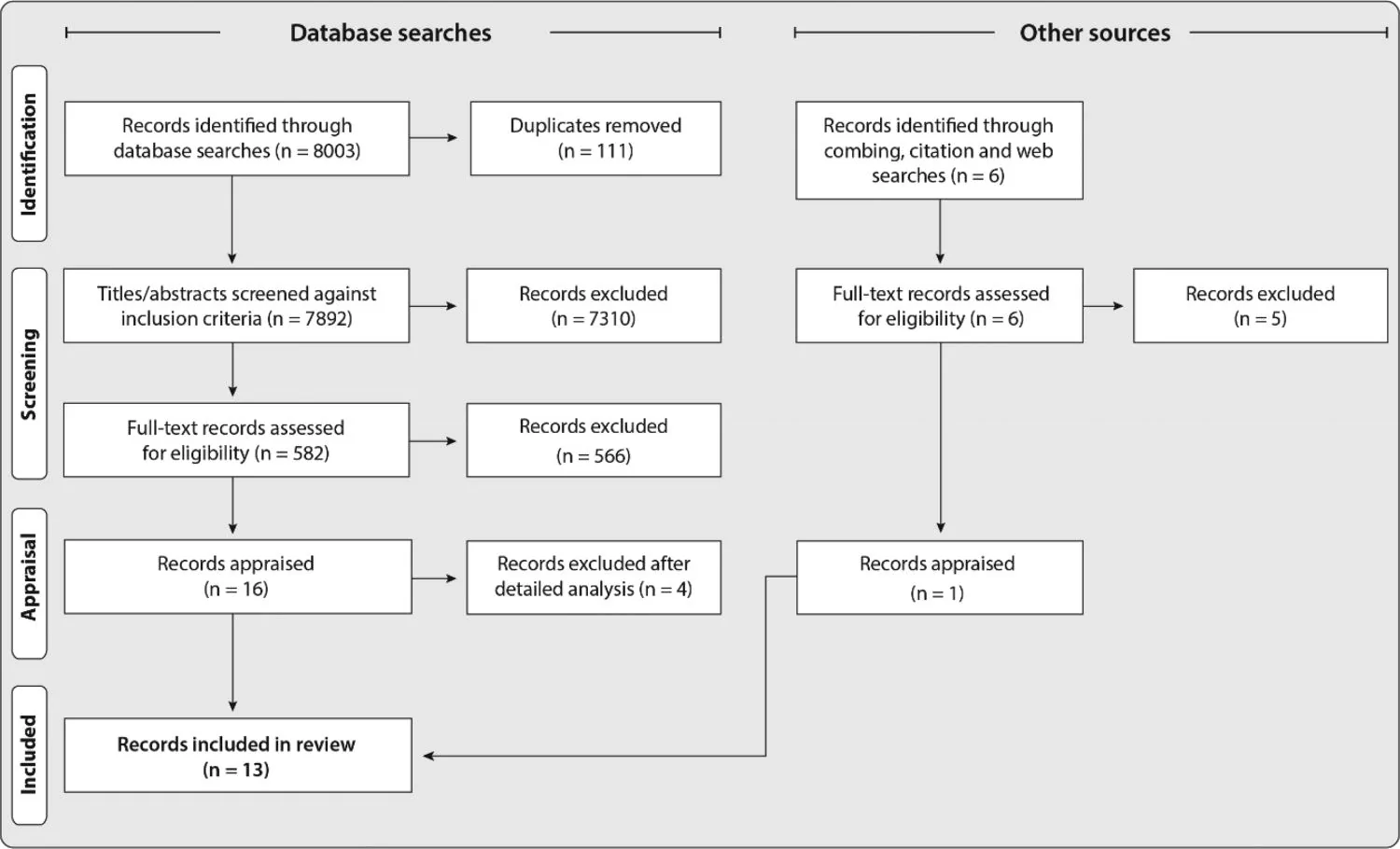
Study selection flowchart.

Data extraction was performed by three researchers (IOW, PM, and MA) iteratively using bespoke templates. Extracted data were appraised using realist principles of *relevance* (usefulness for theory building and testing) and *rigour* (trustworthiness of the methods supporting each specific context-mechanism-outcome (CMO) contribution) [18,19,21,37]. An audit trail of inclusion decisions, theory refinement, and analytic iterations was maintained throughout the review.

A context-mechanism-outcome (CMO) analysis was undertaken for all included articles [18,20,37]. Data were coded deductively against the initial programme theory and inductively for emerging contexts, mechanisms and outcomes [21,22]. CMOs were compared across studies to identify demi-regularities and examine how context shaped mechanisms and outcomes [22,38]. Conflicting evidence was used to refine, split or discard candidate CMOs.

Evidence was grouped by intervention to understand each in context, drawing on included articles, reports, wider networks and discussions with relevant experts [39–47]. Experts were identified through authorship, recommendations and responses from Community Chaplaincy Listening leads [48].

Analysis then became progressively reductive, distilling what worked, for whom and in which contexts. Programme theories were refined through constant comparison with the initial theory and realist logics of inference: juxtaposition, reconciliation, adjudication and consolidation [18,22]. Contradictory evidence, including cultural variation, informed refinements agreed by the research team. CMO frameworks are summarised in Appendix C.

## Results

Thirteen articles met our final inclusion criteria, describing five distinct spiritual health interventions that had taken place in European primary care [11,47,49–59]. (Table 1) The interventions were: 1. Community Chaplaincy Listening (CCL) [49,51,52] and 2. Primary Care Chaplaincy in Scotland [11,50]; 3. Chaplains for Wellbeing in England; 4. Spiritual Caregivers (SCG) in The Netherlands) [47,57], and 5. HoPES3 intervention (Germany) [53–56]. We found multiple training interventions that had not undergone formal trial and evaluation, for example the EMAP (existential communication in general practice) tool in Denmark, [60–62] the Composite Holistic Needs Assessment Adaptive Tool-Prostate (CHAT-P) for prostate cancer in the UK, [63] and the ‘Diamond model’ in Belgium and the Netherlands.[64] Some CFBOs employed ‘parish nurses’ [16,65], however these were excluded because they were not interventions from primary care. We found other interventions that had not been formally evaluated, for example a London chaplaincy project and a Danish ‘inspiration catalogue’ to support spiritual health needs in primary care [66]. The contexts, mechanisms, and outcomes of the five interventions were analysed in terms what works, for whom, and in what contexts. (Appendix C) Analysis of the interventions was then synthesised as a whole, with contexts, mechanisms, and outcomes grouped thematically (Figure 3).

**Figure 3.**
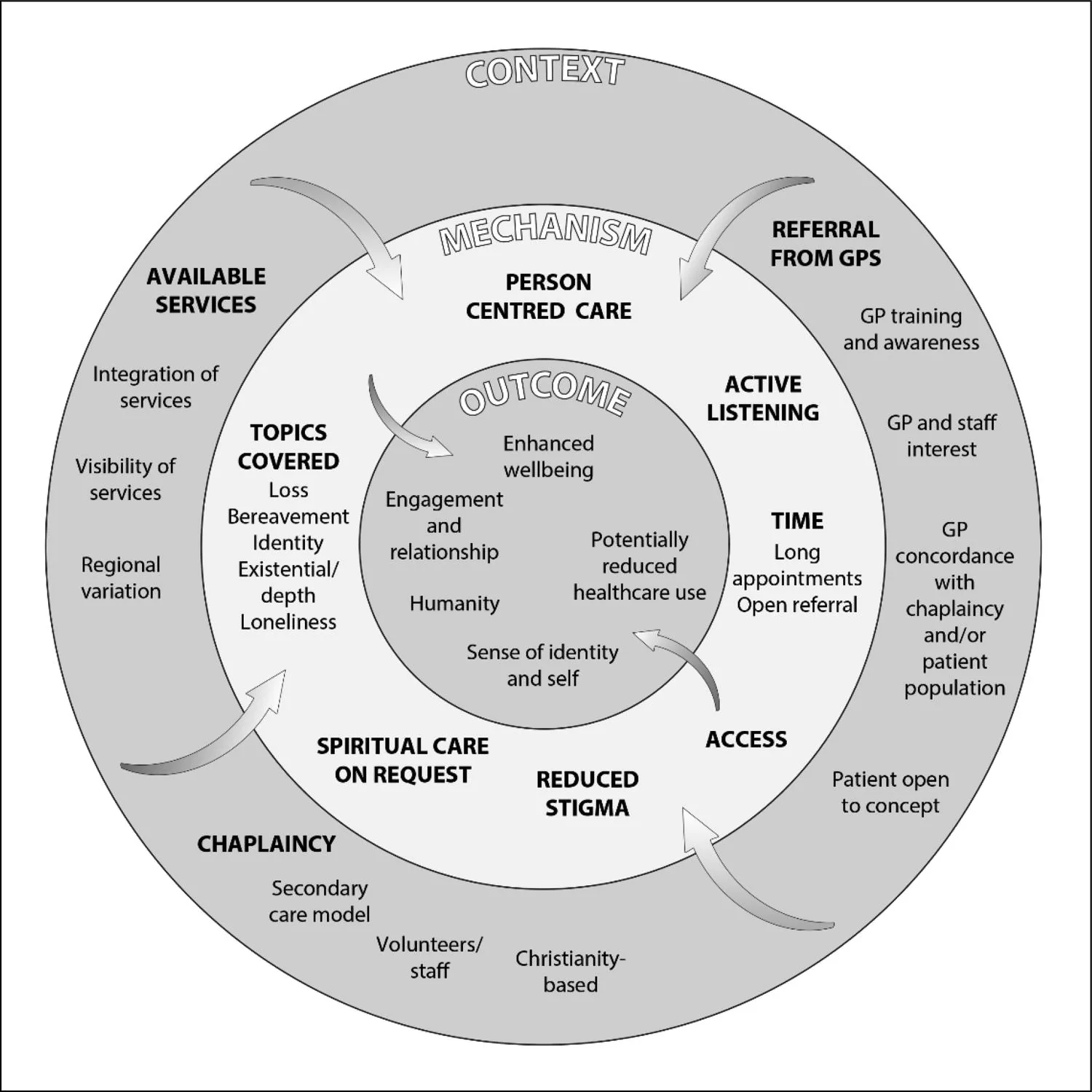
A summary of the programme theory of spiritual health interventions in Europe.

**Table 1.** Document characteristics.

Intervention			Published articles relating to the intervention							
Intervention name	Intervention delivered by	Location	Author / Year	Overview of research	Key features of intervention	Intervention referral route	Intervention dates	Outcome measures	Intervention participant numbers	Intervention participant information (summary)
**Community Chaplaincy Listening (CCL)**	Primary Care Chaplains	Scotland	Mowat, H., et al. (2012)^52^	Data drawn from questionnaire responses and semi-structured interviews with patients, GP referrers, chaplain listeners, and senior health service managers	CCL spiritual listening intervention, introduced into four NHS Health Boards	GP (mostly), other healthcare professional, or self	2010 − 2011	–	49 patients	No demographics
			Bunniss, S., et al. (2013)^51^	Data drawn from descriptive statistics, semi-structured interviews, focus groups, and survey responses	CCL spiritual listening intervention piloted in GP surgeries	Typically by GP	09/2011 − 07/2012	–	250 patients 215 patients seen and discharged 60 patients booked but did not attend	Ages 18-89, largest cohort (41%) aged 40-59. 75% of patients female.
			^Snowden, A., et al. (2023)49^	Prospective, multisite, paired-sample ‘before and after’ study, data drawn from quantitative measures	CCL spiritual listening intervention delivered/embedded in healthcare centres	Most commonly by GP	2018 − 2019	Scottish Patient Reported Outcome Measure (PROM)^1^ Health Related Quality of Life (EQ-5D-3L)^2^	143 patients	100 (70%) female, 43 (30%) male 50% neither religious nor spiritual
**Primary Care Chaplaincy (PCC)**	Primary Care Chaplains	Scotland	^Macdonald, G. W. (2018)11^	A retrospective observational study, data drawn from routinely collected quantitative data	Listening intervention fusing both CCL and biopsychosocial approaches	Referral decision based entirely on clinicians’ consultation	03/2015 − 07/2017	Warwick-Edinburgh Mental Well-Being Scale (WEMWBS)^3^	164 patients	Ages 16-65+, 65% aged over 40,98% white, 80% female, 50% paid employment
			Giffen, S. & Cowey, E. (2020)^50^	Qualitative study, data drawn from semi-structured interviews with GPs exploring reasons for referral		–	01/2018 − 05/2018	–	9 GPs	77% Christian, 67% male
**Chaplains for Wellbeing (C4W)**	Chaplains	England	^Kevern, P. and L. Hill (2015)58^	A retrospective study, data drawn from quantitative measures	Delivered across 134 primary care centres within a Wellbeing Hub model	GP Sandwell Wellbeing Hub, or self	01/2011 − 01/2013	Warwick-Edinburgh Mental Well-Being Scale (WEMWBS)^c^	107 patients	No details of participant demographics included
			^McSherry, W., et al. (2016)59^	Qualitative study, data drawn from semi-structured interviews with patients			03/2014 − 05/2014	–	16 patients	Ages 29-69, 10 (63%) female, 6 (38%) male 12 (75%) Christian, 4 (25%) other faiths
**Spiritual Caregivers (SCG)**	Spiritual Caregivers	Netherlands	^Koper, I., et al. (2019)57^	Mixed methods study, data drawn from questionnaire (structured and open questions) and focus group	Listening intervention, including whole-person care, end of life, meaning-making, existential, relational, and religious issues	Usually clinical staff, e.g. GPs and district nurses, self-referral also available	04/2016 − 08/2016	–	31 spiritual caregivers	Ages 27-74, 22 (71%) female, 9 (29%) male 19 (61%) Christian, 9 (29%) Humanist
			Visser, A. & Zock, H. (2025)^47^	Secondary analysis of mixed methods data drawn from focus groups, questionnaire, and comparative case studies			08/2019 − 02/2021	–	70 chaplains, 38 patients 16 healthcare providers/partnerships	No demographics
**Holistic care program for elderly patients to integrate spiritual needs, social activity and self-care into disease management in primary care (HoPES3)**	GPs and medical assistants	Germany	^Sturm, N., et al. (2022)56^	Non-blinded, exploratory, cluster-randomized, controlled trial, data drawn from quantitative measures	Intervention focused on: spiritual needs, self-care, social activity and loneliness. GPs collected patients’ spiritual histories using SPIR tool, medical assistants provided information about social activities and non-pharmacological interventions	GPs assessed for eligibility from those already engaged in disease management programme	03/2019 − 06/2020	Self-Efficacy for Managing Chronic Disease 6-Item Scale (SES6G)^4^ 12 Item Short-Form Health Survey (SF12)^5^	164 patients in intervention group	Ages 70-91, 87 (53%) female, 16 (10%), university degree, 136 (87.2%) Christian
			Mächler, R., et al. (2022)^55^	Mixed methods study on spiritual history-taking, data drawn from questionnaire and interviews with patients			02/2020-06/2020	–	29 patients in interview group	Ages 70-85, 13 (44%) female, 6 (21%) university degree, 23 (85%) Christian
			Mächler, R., et al. (2023)^54^	Qualitative study, data drawn from semi-structured interviews with patients exploring social aspect of spiritual history intervention						
			^Huperz, C., et al. (2023)53^	Mixed methods study on spiritual history-taking, data drawn from questionnaire and interviews with GPs and medical assistants (MAs)			07/2019-06/2020	–	11 GPs and 12 MAs in interview group	GPs: ages 36-64, 6 (55%) female, 6 (55%) Christian. MAs: ages 20-55, 12 (100%) female, 11 (92%) Christian

^1^ Snowden, A., Tan, H., & Karimi, L. (2021). Statistical fit is like beauty: A rasch and factor analysis of the Scottish PROM. Journal of Health Care Chaplaincy.

^2^ EuroQol Research Foundation (2018) EQ-5D-3L User Guide. https://euroqol.org/publications/user-guides (Accessed: 16/06/2025).

^3^ Tennant, R., Hiller, L., Fishwick, R., Platt, S., Joseph, S., Weich, S., Parkinson, J., Secker, J. and Stewart-Brown, S. (2007) ‘The Warwick-Edinburgh Mental Well-being Scale (WEMWBS): development and UK validation’, Health and Quality of Life Outcomes, 5(1), p. 63.

^4^ Freund, T., Gensichen, J., Goetz, K., Szecsenyi, J. and Mahler, C. (2013) ‘Evaluating self‐efficacy for managing chronic disease: psychometric properties of the six‐item Self‐Efficacy Scale in Germany’, Journal of evaluation in clinical practice, 19(1), pp. 39-43.

^5^ Ware, J.E., Kosinski, M. and Keller, S.D. (1996) ‘A 12-Item Short-Form Health Survey: construction of scales and preliminary tests of reliability and validity’, Medical care, 34(3), pp. 220-233.

All five interventions relied on General Practitioners (GPs) or other clinical staff to identify patients who might benefit, either ad hoc [11,47,51,57,58] or during planned chronic illness reviews. [70 Referral therefore depended on clinicians recognising spiritual need and knowing local pathways. Although services claimed to support people of “all faiths and none”, this was not formally evaluated, nor was the effect of cultural, religious or ethnic concordance between patients and practitioners.

. Patient demographics were poorly reported. No intervention reported ethnicity, all involved adults, and one was restricted to those aged over 70 [56]. Staff in UK and German interventions appeared predominantly Christian, whereas Dutch spiritual caregivers included Christian, Humanist, Buddhist, Islamic, Hindu, Jewish and non-religious backgrounds [47,57].

Some UK chaplains were embedded within primary care multidisciplinary teams [49,50]. Their role overlapped with social prescribing, (Figure 4) although chaplains explicitly addressed spiritual concerns. No study compared spiritual caregiving with other primary care roles. Services often depended on a small number of motivated individuals and could decline or end when they left [49]. Sustainability was further limited by short-term funding [47,56,57] and reliance on volunteers [52].

**Figure 4.**
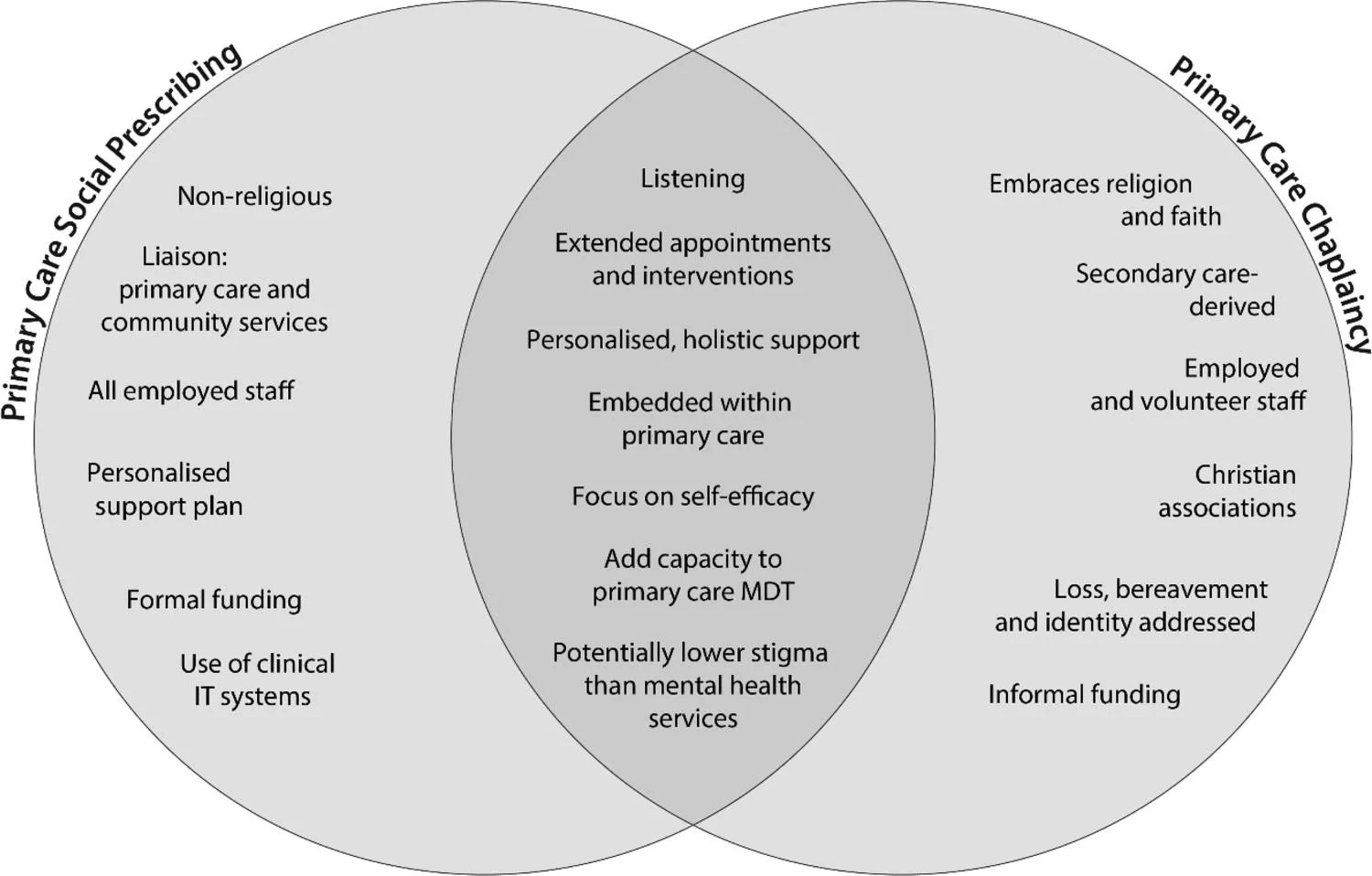
The overlap and differences between chaplaincy and social prescribing within primary care. MDT: Multi-Disciplinary Team.

Interventions offered speedy access, person-centred listening, longer appointments and support with existential concerns, loss, identity and religion, including prayer when requested. Patients who are religious or spiritual may be more likely to benefit from these interventions [56], however other studies identified benefits for people who did not consider themselves religious, or spiritual [50]. Reported outcomes included improved patient wellbeing and patient-practitioner relationships [11,49–59], reduced GP attendance [11] and possibly reduced prescribing [52]. However, general health measures did not clearly distinguish spiritual health from mental wellbeing. The Scottish PROM, designed for spiritual care, showed benefit in over half of CCL participants.

Potential harms, such as religious trauma, were rarely examined. One study reported potentially harmful medical advice from a spiritual caregiver [47], and 2.5% of HoPES3 participants found the intervention stressful [56]. While those providing chaplaincy support assert that they provide care to patients of “all faiths and none”[67], interfaith and cross-cultural experiences were not evaluated.

Four of the interventions used validated Patient Reported Outcome Measures (PROMS) to report outcomes from the interventions. The PROMS used were the Scottish Patient Reported Outcome Measure, [49] Warwick-Edinburgh Mental Well-Being Scale (WEMWBS) [11,50,58], the Self-Efficacy for Managing Chronic Disease 6-Item Scale (SES6G) [56], the Short-Form 12 Health Survey [56], and the Health Related Quality of Life (EQ-5D-3L) [49]. These measures assessed general wellbeing, health and/or quality of life. No validated spiritual health PROMs were used (for example the Functional Assessment of Chronic Illness Therapy - Spiritual Well-Being [68]) although existential aspects were included in the Scottish PROM [49]. The five interventions found beneficial outcomes in terms of improvements in emotional well-being, self-efficacy, mental health, and patient–provider relationships. The ED-5D-3L appeared to be less sensitive to spiritual shifts in outcome, despite improvements in health in other areas measured.

Interventions appeared most effective for adults with emotional, existential or spiritual distress when trained practitioners offered time, attentive listening and person-centred support. Success depended on clinicians recognising spiritual need, accessible referral pathways, stable funding, multidisciplinary integration and clear role boundaries. Benefits were reported across religious and non-religious patients, although evaluation was limited. Local dependence, short-term funding and limited cultural or religious concordance remained important vulnerabilities.

## Discussion

### Main findings

This review identified five published interventions to improve spiritual health in primary care across England, Scotland, Germany and the Netherlands [11,49–59]. The German HoPES3 project, which targeted adults over 75 in one region. This short-term initiative sought to address the spiritual needs of older people and demonstrated feasibility within a specific demographic, though its restricted scope limited broader applicability. The three UK based projects were chaplaincy-based services, rooted in Christian traditions, and sustained by highly motivated individuals [11,49–52,59], resulting in uneven access. Outcomes were generally positive, with patients reporting benefits, improved practitioner relationships, and reduced GP attendance. Comparisons across interventions were limited because studies assessed different constructs using measures of mental wellbeing, self-efficacy, general health and health-related quality of life, alongside service-use data and qualitative accounts of benefit. The Scottish PROM included existential dimensions, but none of the interventions used a validated measure specifically designed to assess spiritual health as a separate aspect of health. The Netherlands offered the most integrated model, supported by a legal right to spiritual care and state-funded Humanist chaplaincy [69]. However, no model demonstrated sustained or equitable scalability for Europe. Outside the Netherlands, services often relied on short-term funding, volunteers and a few key individuals. Evidence on equality of access, population reach and whether services met the needs of culturally and religiously diverse groups was limited.

Chaplaincy appears as the dominant model and retained strong links to religious traditions, despite expansion to include Humanists and “spiritual care professionals”[42,43,45–47]. Claims to support people of “all faiths and none” were not formally evaluated, and non-Christian chaplains were under-represented [43]. Hospitals may sustain multifaith chaplaincy through economies of scale that are unavailable to smaller primary care organisations [45,70]. Strengths included person-centred care, active listening and willingness to address existential concerns. Stakeholders suggested that chaplaincy may be more acceptable than talking therapies for some patients, although this was not evaluated. Outcomes were also rarely compared with other holistic or personalised approaches, such as social prescribing, counselling, health and wellbeing coaching, Positive Health or supported self-management [71–73].

Overall, the evidence shows both potential benefits and substantial gaps in European spiritual health care. Outcomes depended heavily on context. Important mechanisms included time-rich relational encounters, skilled listening and permission to explore existential concerns. Interventions benefitted adults with recognised spiritual needs where trained staff and stable structures were available, but were unsustainable when provision was patchy, under-resourced or limited to one faith tradition. UK provision remains fragmented, reliant on individuals and far below the estimated need for 1,335 full-time chaplains [41]. The Netherlands offers the most embedded model, while Germany demonstrates targeted feasibility. As yet, no country has achieved an equitable, sustainable approach to spiritual health in primary care.

### Comparison with other literature

To our knowledge, this is the first realist review of spiritual health interventions in European primary care. A recent scoping review examined chaplaincy in outpatient settings such as oncology and palliative care [44], but these differ substantially from primary care. Previous reviews have considered spiritual health within GP consultations [31,74], while noting that GPs may lack the skills or inclination to act as spiritual caregivers [75].

Related approaches, including green prescribing [76], forest bathing [77], and ‘blue prescribing’ (water based intervention) [78] may also support connection with self, nature and the wider world, although they are not usually framed explicitly as spiritual health interventions. In secular societies, such approaches may complement explicitly spiritual models.

Parish nursing shows some willingness among community spiritual organisations to work with primary care [79–81], but we found little evidence of sustained formal collaboration. Social prescribing may provide a bridge to these community assets. The UK based Theos think tank has highlighted the potential role of community faith-based organisations (CFBOs) in social prescribing [82], and their value to global healthcare is recognised [83,84], though often in terms of practical resources rather than spiritual health [82,83,85]. Despite calls to embed spiritual care within primary care [10], there is little evidence of this being achieved in a sustainable way.

### Strengths and limitations of this study

This realist review examined interventions within their real-world contexts, enabling consistent analysis of contexts, mechanisms and outcomes. Restricting the review to Europe supported comparison across broadly similar cultural settings and reflected the team’s expertise, but excluded potentially relevant approaches elsewhere.

Related interventions, including green and blue prescribing, were excluded because they were framed in terms of wellbeing or general health rather than spirituality, despite addressing connection with nature and the wider world [77,78,86,87]. This may reflect stigma around spiritual language and assumptions about religiosity [88–91]. While a ‘working definition’ of spiritual health was used by the team to ensure meaning- deliberately broad search terms, including wellbeing, holistic health and salutogenesis, helped identify studies not explicitly labelled as spiritual health. Studies where the authors identified a spiritual aspect were included without consideration of the team’s working definition, as the authors’ definitions took precedence in those cases. This breadth was a strength of the review. Spiritual health overlaps with, but remains distinct from, mental health and wellbeing and therefore may therefore still be overlooked in the literature.

### Implications for policy, practice, and research

This review highlights both the promise and the limitations of existing approaches to spiritual health in primary care. A universal chaplaincy model would require substantial investment, and may unnecessarily duplicate services already in place, such as social prescribing, parish nursing, and work already underway by CFBOs. Evaluations of existing interventions are inconsistent, with outcomes often framed in terms of general wellbeing rather than distinct spiritual health benefits, such as improved meaning or purpose, reduced existential distress, strengthened connection, or greater ability to live in accordance with personal values and beliefs. This makes it difficult to determine whether these interventions offer benefits beyond those provided by other holistic or relational services, and therefore to establish their unique value for commissioners.

Chaplaincy’s Christian heritage may limit its relevance to wider society, particularly where minority faith populations are too small to sustain dedicated provision. Although the Netherlands offers a more pluralist, legislatively supported model, no European country has established an equitable and sustainable primary care service. This raises questions about whether even multifaith chaplaincy is the most appropriate model for diverse, pressured primary care systems, where duplication of existing roles should be avoided. Replicating secondary care chaplaincy at scale is unlikely to be feasible without substantial investment.

An alternative is to build on existing social and religious capital. Community faith-based and non-faith organisations already provide pastoral and spiritual support, while UK social prescribing offers an established link between primary care and community services. Primary care staff could identify spiritual needs and connect patients with appropriate local provision, creating a more sustainable, inclusive and context-sensitive model. This would reduce dependence on isolated individuals, address inequities in access and make use of existing community assets. As connector roles become embedded in UK primary care and guidance develops for social prescribers and faith groups, spiritual care could be reframed as a community-linked, asset-based approach rather than remaining confined to small, underfunded pockets of primary care chaplaincy.

## Conclusion

This realist review found isolated examples of motivated teams providing spiritual health care in primary care, but no standardised or consistently evaluated approach. Sustainability was limited by short-term funding, local dependence, and services that were not fully embedded within primary care. The Netherlands was the only context with a formal right to spiritual care, and therefore the most integrated model. Primary care chaplaincy was valued where available, but not universally accessible or suited to all patients. A realist perspective highlights that what works is highly context dependent, shaped by local infrastructure, practitioner availability, and cultural fit. Outcome measures were inconsistent and tended to focus on wider, sometimes indistinct dimensions of wellbeing rather than clearly defined spiritual health outcomes. Upskilling primary care staff to recognise spiritual needs and link patients with community faith and non-faith organisations, including through social prescribing, may offer a more inclusive and sustainable option. Future research should examine whether chaplaincy or alternative, asset-based models can provide equitable, long-term spiritual health support across larger populations, and whether chaplaincy genuinely meets the needs of all faiths and none.

## Use of generative artificial intelligence

Artificial intelligence (ChatGPT, OpenAI GPT-5.1, 2025) was used at a final proof reading stage to support editing and refinement of academic writing, including improving clarity, grammar, and structure. All content, interpretations, and final decisions were made by the authors, who take full responsibility for the accuracy and integrity of the manuscript.

## Supplementary Material

Supplemental Material

Supplemental Material

Supplemental Material
