# Supplementary material for: What has been used to improve patients’ spiritual health in primary care: A realist review
Source: Eur J Gen Pract. 2026 Oct 12;32(1):2739239. doi: 10.1080/13814788.2026.2739239 (PMC13660543; doi:10.1080/13814788.2026.2739239)
Supplement: Supplementary file 2 [file IGEN_A_2739239_SM9696.docx]

Appendix B Full database search strategies

**Database(s): Ovid MEDLINE(R) ALL 1946 to June 24, 2025 – Dates of Search 24/05/2024 and updated 25/06/25**
Search Strategy:

| **#** | **Searches** |
| --- | --- |
| 1 | exp religious personnel/ |
| 2 | pastoral care/ |
| 3 | spiritual care/ |
| 4 | holistic care/ |
| 5 | holistic nursing/ |
| 6 | (chaplain* or priest* or pastor? or imam? or rabbi? or humanis? or social prescriber* or care navigator*).kf,tw. |
| 7 | (spiritual or faith? or faith based or holistic).kf,tw. |
| 8 | 1 or 2 or 3 or 4 or 5 or 6 or 7 |
| 9 | exp primary health care/ |
| 10 | exp general practice/ |
| 11 | general practitioner/ |
| 12 | ((primary or general or family) adj2 (practice or physician)).kf,tw. |
| 13 | (primary care or primary health).af. |
| 14 | 9 or 10 or 11 or 12 or 13 |
| 15 | 8 and 14 |
| 16 | limit 15 to yr="2000 -Current" |

**Database(s): Embase 1974 to 2025 June 24 – Date of search 25/06/2025**
Search Strategy:

| **#** | **Searches** |
| --- | --- |
| 1 | exp religious personnel/ |
| 2 | pastoral care/ |
| 3 | spiritual care/ |
| 4 | holistic care/ |
| 5 | holistic nursing/ |
| 6 | (chaplain* or priest* or pastor? or imam? or rabbi? or humanis? or social prescriber* or care navigator*).tw. |
| 7 | (spiritual or faith? or faith based or holistic).tw. |
| 8 | 1 or 2 or 3 or 4 or 5 or 6 or 7 |
| 9 | exp primary health care/ |
| 10 | exp general practice/ |
| 11 | general practitioner/ |
| 12 | ((primary or general or family) adj2 (practi* or physician*)).tw. |
| 13 | (primary care or primary health*).tw. |
| 14 | 9 or 10 or 11 or 12 or 13 |
| 15 | 8 and 14 |
| 16 | limit 15 to yr="2000 -Current" |

**Database(s): APA PsycInfo 1806 to June 2025 Week 3 – Date of Search 25/06/25**
Search Strategy:

| **#** | **Searches** |
| --- | --- |
| 1 | exp religious personnel/ |
| 2 | exp pastoral counseling/ |
| 3 | exp spiritual care/ |
| 4 | exp holistic health/ |
| 5 | exp spirituality/ |
| 6 | (chaplain* or priest* or pastor? or imam? or rabbi? or humanis? or social prescriber* or care navigator*).tw. |
| 7 | (spiritual or faith? or faith based or holistic).tw. |
| 8 | 1 or 2 or 3 or 4 or 5 or 6 or 7 |
| 9 | exp primary health care/ |
| 10 | exp general practitioners/ |
| 11 | ((primary or general or family) adj2 (practi* or physician*)).tw. |
| 12 | (primary care or primary health*).tw. |
| 13 | 9 or 10 or 11 or 12 |
| 14 | 8 and 13 |
| 15 | Limit 14 to yr="2000 -Current" |

**CINAHL SEARCH – Date of Search 25/06/2025**

Search: ((TI (chaplain* or priest* or pastor? or imam? or rabbi? or humanis? or social prescriber* or care navigator* or spirituality or religion or faith or belief system or spiritual or faith based or holistic) OR AB (chaplain* or priest* or pastor? or imam? or rabbi? or humanis? or social prescriber* or care navigator* or spirituality or religion or faith or belief system or spiritual or faith based or holistic) OR (MH "Holistic Care" OR MH "Holistic Health" OR MH "Holistic Nursing" OR MH "Religious Personnel+")) AND (TI (primary care or primary health care or primary healthcare or general practitioner or gp or family doctor or primary care or family physician or general physician) OR AB (primary care or primary health care or primary healthcare or general practitioner or gp or family doctor or primary care or family physician or general physician) OR (MH "Primary Health Care" OR MH "Family Practice" OR MH "Physicians, Family")))Filter dated 01/01/2020 to 06/25/2025
