# Supplementary material for: What has been used to improve patients’ spiritual health in primary care: A realist review
Source: Eur J Gen Pract. 2026 Oct 12;32(1):2739239. doi: 10.1080/13814788.2026.2739239 (PMC13660543; doi:10.1080/13814788.2026.2739239)
Supplement: Supplementary file 3 [file IGEN_A_2739239_SM9695.doc]

**Appendix C- Context Mechanism Outcome analysis**

**Chaplains for Wellbeing (grouped)**

| **WHAT WORKS** | | | **WHAT DOESN’T WORK** | **COMMENTS ON QUALITY** |
| --- | --- | --- | --- | --- |
| **CONTEXT** | **MECHANISM** | **OUTCOME** | **CONTRADICTORY EVIDENCE** |  |
| Patients identified and referred into chaplaincy by GPs (and therefore GP felt could benefit from chaplaincy) and accepted referral. Referrals came from 134 primary care centres. Unclear what the other route in was (12 out of 16 in McSherry were referred, the route of the remaining 4 was not specified.)  16 working age patients, 12 Christian  Referrals within the context of loss, bereavement, and challenge to identity  Delivered within a deprived local authority area | Time – long appointments over months or years  Chaplain role broad – 8 key functions described which were not mutually exclusive.  Active listening – coaching (evoking rather than advising) – non-judgemental – person-centred – validating  Focus on wellbeing rather than contested terms (e.g. spirituality, religion)  A designated calm space to focus on the self  Patient dignity was preserved  Prayer was offered – ‘most reported positively’, however** | Improved wellbeing (WEMWBS) - improvement in scores highest for those with initially lower levels of wellbeing. Improvements were not dependent on age, sex, ethnicity or number of visits.  Develop strategies to face and rebuild confidence.  Improved sense of identity and self.  No mention of cost-effectiveness or use of GP time ongoing. | Chaplaincy includes the ‘historical association between church and state’ - the ‘secular credentials’ of the NHS act as a block to implementation.  WEMWBS tool not employed consistently. This apparently reduces danger of falsely low initial readings from acute distress – however acute distress is still a legitimate reading and that reading should not be considered false. E.g. resolving mental health crisis does not mean that the low where the crisis occurred was not relevant to the individual’s wellbeing. | **Qualitative and quantitative evaluation of an intervention delivered within an area of deprivation, in which the quantitative element contained correlation between the sex, ethnicity and employment characteristics of the sample and population under study.**  While ethnicity was acknowledged –its intersection with religion may be important here and was not explored.  WEMWBS (as with wellbeing measures in general) are ‘muddy’ – and being delivered by Chaplains could introduce additional bias.  Study made no mention of limitations. (There were – incorrectly placed in the conclusions of Kevern and Hill.)  Breadth and vagueness of key functions make accurate causal classification of mechanism and outcomes challenging. This is compounded by lack of control over other variables.  ** this comment about prayer was drawn from interviews with the 16 out of the 20 patients (from the 108 purposively sampled – assumed from the 107 included in Kevern - however not stated) who responded to written information sheets. |

**Community Chaplaincy Listening (grouped)**

| **WHAT WORKS** | | | **WHAT DOESN’T WORK** | **COMMENTS ON QUALITY** |
| --- | --- | --- | --- | --- |
| **CONTEXT** | **MECHANISM** | **OUTCOME** | **CONTRADICTORY EVIDENCE** |  |
| Support from wider health structures (Scottish Health Boards, NHS Education for Scotland)  Referral from GP (relies on GP endorsement of patient suitability and of CCL service, remembering to refer)  Embedded into primary care (rather than ‘squeezed in’)  Referrals within the context of loss, search for meaning, and shock | Listening  Long appointment times length of intervention, open-ended intervention  Swift access – short/no waiting lists  Secular rather than religious  Less stigma at referral than for psychiatry | PROM higher, however EQ-5D fell**  Improved spiritual health – reduced anxiety, more in control, greater sense of peace.  Reduced medical prescribing? (qualitative outcome)  Shifted perspective, found purpose, enhanced wellbeing, develop coping strategies | HCPs not viewing the model as relevant or valuable  Lack of communication between GPs and chaplains | Outcomes, e.g. ‘patients overwhelmingly reported having a positive experience with the CCL service’ drawn from limited sample – i.e. 2 interviews and 16 questionnaires.  CCL1 data drawn from: 16 interviews [patients (n=2)] and 26 questionnaire responses [patients (n= 16)]  No demographics or discussion of sampling – some participants included in both interviews and questionnaires.  Reports based on data collected on limited % of those receiving intervention (CCL1 2/49 interviewed, 16/49 responded to questionnaires; CCL2 18/250 interviewed).  **Unexpected finding – improvement with PROM but not EQ-5D-3L… no clear reason for this.  Average attendance of three sessions is low. High DNA rate for first appointment (19%) not explored – what are the reasons for this / problems with the service? |

# HoPE3 (grouped)

| **WHAT WORKS** | | | **WHAT DOESN’T WORK** | **COMMENTS ON QUALITY** |
| --- | --- | --- | --- | --- |
| **CONTEXT** | **MECHANISM** | **OUTCOME** | **CONTRADICTORY EVIDENCE** |  |
| Primary care setting with multi-morbid patients already engaged in a wider health programme.  Intervention aimed to improve self-efficacy – referrals were skewed towards those who GPs felt would benefit from the intervention.  Assumed that patients had access to the internet, and local community resources.  Concordance of attitudes towards spirituality between patients and practitioners. | Conversations with non-clinical staff  Time allocated to conversations  Framing conversations about decisive life events and personal sources of strength (as opposed to SH) | Improvement in mental wellbeing  Improved patient-practitioner relationship | No relevant effect on primary outcome (improved self-efficacy)  Terminology around spirituality for non-spiritual patients was confounded with religion  Sticking to a ‘script’ when delivering SPIR (as opposed to more fluid, natural conversation) | **Improvement in mental wellbeing identified in those who were connected socially and spiritually at the start – and those already engaged in wider programme of health - thus potentially contributing to a widening of health inequity.**  Given the concordance, outcomes are perhaps only relevant to older Christian patients (assumed White, given lack of data around ethnicity?)  Quality of delivery of intervention may depend on the attitude/ability of the practitioner.  Would GPs have time to deliver SPIR with less healthy patients? |

# Primary Care Chaplaincy (grouped)

| **WHAT WORKS** | | | **WHAT DOESN’T WORK** | **COMMENTS ON QUALITY** |
| --- | --- | --- | --- | --- |
| **CONTEXT** | **MECHANISM** | **OUTCOME** | **CONTRADICTORY EVIDENCE** |  |
| Embedded into primary care  SH positioned as ‘within’ MH provision  SH valued by clinicians  Engagement with intervention depends primarily on GP referrals | Time - Long appointment times, length of intervention, open-ended intervention  Prompt access  Listening | Improved wellbeing (WEMWBS)  Reduction in GP appts for those not prescribed AD | Dependent on GPs feeling ‘comfortable’ referring | More robust statistical analysis (Macdonald)  Interviews with GPs (Giffen & Cowey) have no outcome measure  Reduction in GP appointments is a ‘muddy’ measure – e.g. patients prescribed AS will require GP supervision, partly explaining lower number of GP appointments in those attending PCC not on AD.  Giffen & McDonald – no dates, numbers of responses – descriptive report |

# Spiritual Caregivers

| **WHAT WORKS** | | | **WHAT DOESN’T WORK** | **COMMENTS ON QUALITY** |
| --- | --- | --- | --- | --- |
| **CONTEXT** | **MECHANISM** | **OUTCOME** | **CONTRADICTORY EVIDENCE** |  |
| Service delivery is shaped by shifting commissioning pressures that favour measurable outcomes over whole-person care. Although the Dutch SCG model is more inclusive of non-Christian, Humanist, and non-religious providers than UK chaplaincy, a Christian slant remains evident, and most patients(60%) preferred a caregiver with a similar religious background or worldview. | Universal, age-inclusive spiritual care, with some caregivers specialising in specific life or medical contexts. SCGs focus on holistic support for existential, relational, and religious concerns, and are viewed as distinct from other health professionals through their emphasis on meaning-making and “life questions” counselling. Their services are valued but rely on complex, tiered funding and sometimes involve participation in multidisciplinary discussions. | Implicit assumption that the delivery of a SH / listening intervention leads to a positive outcome, not formally assessed | Funding constraints and organisational structures limit the reach of spiritual care, with chaplains expected to meet externally set priorities while lacking the “entrepreneurial” capacity to secure resources. Referral pathways remain weak: practitioners often need repeated reminders about SCG roles, and perceptions of their closeness to organised religion can reduce referrals. Although services claim to support “all faiths and none,” financial incentives sometimes restrict provision to specific cohorts, and registration requirements can exclude vulnerable groups such as migrants and unhoused people. | *Geestelijk verzorger* (Dutch term for SCG) does not have direct translation to English, can be either SCG and/or healthcare chaplain but without association to Christianity that chaplain has in the UK.  Data collection took place when the new government subsidy scheme was being implemented. Some chaplains would provide care for free, outside of funding remit- ?sustainable?  Lack of outcome measure.  There are multiple possible explanations for why some non-Christian SCGs have been posited as supporting mostly people from their own religion. |
